# Functional Expression of Human NKCC1 from a Synthetic Cassette-Based cDNA: Introduction of Extracellular Epitope Tags and Removal of Cysteines

**DOI:** 10.1371/journal.pone.0082060

**Published:** 2013-12-05

**Authors:** Suma Somasekharan, Michelle Y. Monette, Biff Forbush

**Affiliations:** Department of Cellular and Molecular Physiology, Yale School of Medicine, New Haven, Connecticut, United States of America; Albert Einstein College of Medicine, United States of America

## Abstract

The Na-K-Cl cotransporter (NKCC) couples the movement of Na^+^, K^+^, and Cl^−^ ions across the plasma membrane of most animal cells and thus plays a central role in cellular homeostasis and human physiology. In order to study the structure, function, and regulation of NKCC1 we have engineered a synthetic cDNA encoding the transporter with 30 unique silent restriction sites throughout the open reading frame, and with N-terminal 3xFlag and YFP tags. We show that the novel cDNA is appropriately expressed in HEK-293 cells and that the YFP-tag does not alter the transport function of the protein. Utilizing the Cl^−^ -sensing capability of YFP, we demonstrate a sensitive assay of Na-K-Cl cotransport activity that measures normal cotransport activity in a fully activated transporter. In addition we present three newly developed epitope tags for NKCC1 all of which can be detected from outside of the cell, one of which is very efficiently delivered to the plasma membrane. Finally, we have characterized cysteine mutants of NKCC1 and found that whereas many useful combinations of cysteine mutations are tolerated by the biosynthetic machinery, the fully “cys-less” NKCC1 is retained in the endoplasmic reticulum. Together these advances are expected to greatly assist future studies of NKCC1.

## Introduction

Na-K-Cl cotransporters (NKCCs) are membrane transport proteins that mediate coordinated movements of Na^+^, K^+^ and Cl^−^ across the cell membrane. NKCC1 is involved in regulation of cellular Cl^−^ concentration and resting membrane potential and is especially prominent in Cl^−^ secretory epithelia as a critical part of the salt secretion mechanism [1]. NKCC2 is specifically localized in the apical membrane in the thick ascending loop of Henle where it is responsible for a major fraction of salt reabsorption by the mammalian kidney and is inhibited by the clinically important loop diuretic drugs. Because of their central role in electrolyte balance, these transporters are key elements in the control of blood pressure and are therapeutic targets in the management of hypertension [2], [3].

Current studies of the structure, function and regulation of NKCCs and other transporters rely heavily on site-directed mutagenesis to engineer mutant transporters. Although numerous recent advances have made mutagenesis techniques straightforward, they are still somewhat cumbersome where multiple mutagenesis sites are involved, and in any case may involve additional subcloning steps or extensive sequencing where a large cDNA such as NKCC is involved. A very useful approach involves engineering of the target gene of interest to create unique restriction sites throughout the coding region in order to facilitate simple subcloning strategies.

In the present report we describe functional expression of hNKCC1 in HEK cells from a fully synthetic cDNA. The cDNA sequence is designed to silently eliminate most of the pre-existing restriction sites and to include 30 unique silent restriction sites at intervals of approximately 150 bp throughout the open reading frame, as well as convenient sites for insertion of N-terminal tags and for vector insertion. We show that this hNKCC1 is expressed similarly to the native sequence and as expected the transport characteristics are identical.

Using the novel NKCC1 construct, we report a number of important advances in technique and results with human NKCC1. 1) We show that a phospho-dead construct with 15 threonine and serine mutations in the phosphoregulatory domain is expressed at the membrane in HEK cells but can not be activated. 2) We demonstrate that a Cl^−^-sensing YFP tag on NKCC1 can be used as a simple and sensitive assay for NKCC1 activity, readily adaptable to high-throughput methodology. 3) We show that appropriate insertion of epitope tags in extracellular loops is tolerated by the biosynthetic and trafficking machinery, and that the tagged NKCC1s are sensitively detected from the outside of the cell. 4) We evaluate cysteine removal from NKCC1 sequence and find that while the completely “cys-less” construct is not trafficked to the membrane, combinations of “extracellular cys-less”, “TM cys-less” and “intracellular cys-less” are delivered to the membrane and are functional. Together these advances will be of great importance in future studies of NKCC structure and function and in the development of therapeutic agents directed to these critically important transporters.

## Materials and Methods

### Design of synthetic cDNA for hNKCC1

**Figure 1:**
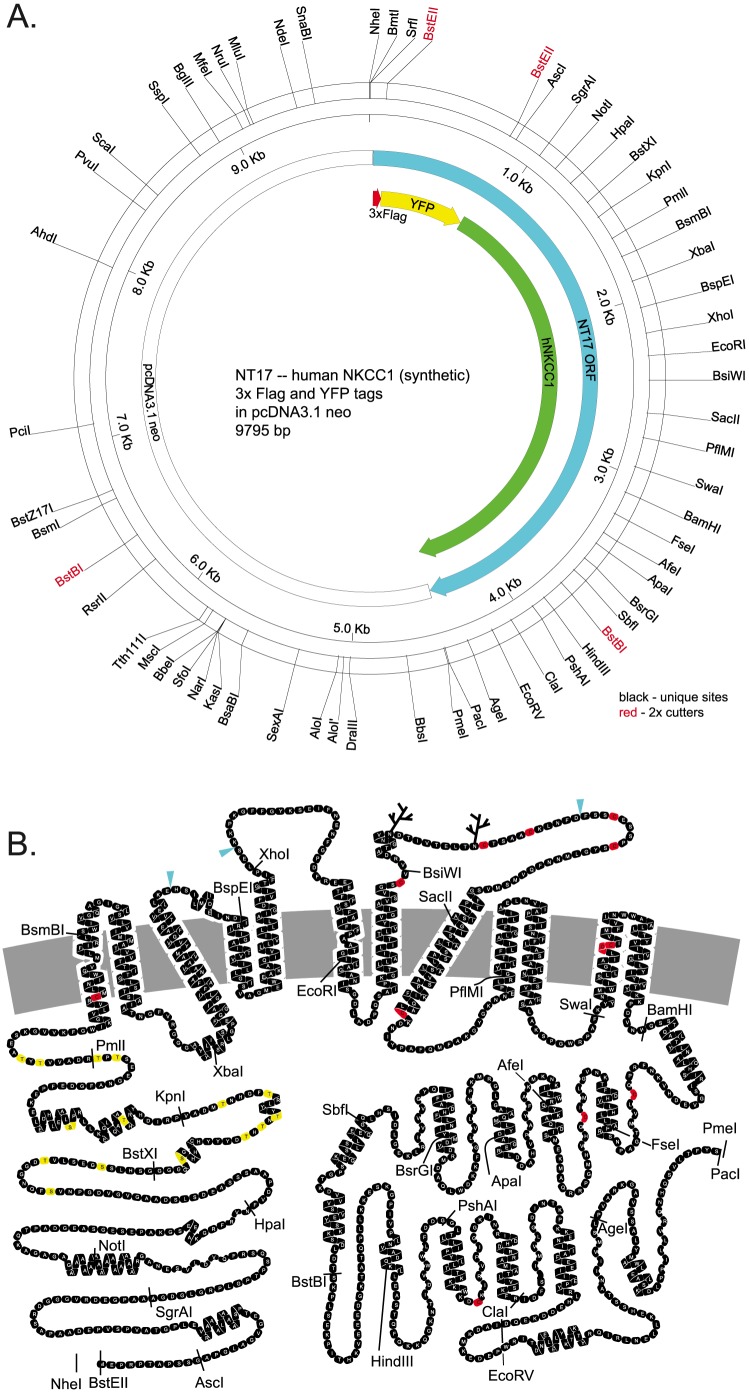
Synthetic human NKCC1 tagged with 3xflag and YFP (NT17). A. Restriction enzyme map showing silent restriction sites. B. 2-D cartoon of NKCC1 protein structure, identifying locations of restriction enzyme cleavage in the corresponding cDNA. Native cysteine residues (red), threonines and serines in the phosphoregulatory domain (yellow) and extracellular epitope insertion sites (blue triangles) are discussed in the text.

To select an optimal set of silent restriction sites in human NKCC1 (fig. 1A) we proceeded as follows: All possible silent restriction sites were determined for the hNKCC1 protein sequence using WebCutter, selecting a subset favoring enzymes that do not cut in our cloning and expression vectors (see below), and ignoring enzymes with unfavorable re-ligation characteristics. All possible sites were plotted graphically, and site selection was hand optimized within the constraints of available possibilities and optimal spacing. It would be desirable to choose sites such that a “cassette” includes a region of interest in the protein (eg. a TM, fig. 1B) – such placement accuracy is simply not practical within the constraints of available silent sites. A number of restriction sites were included in the hNKCC1 and fluorescent protein sequences for diagnostic use, notably SpeI and EagI, also BspHI, MscI, SalI, BbsI, BsaI, AvrII, AclI. After selection of sites, the sequence of the native hNKCC1 was modified to introduce the silent restriction sites, and to silently eliminate pre-existing sites. Where possible in making base changes, optimal human codon preference was used and repetitive sequences were avoided.

At the N-terminus, NheI, XmaI and SrfI cloning sites are followed by a strong Kozak consensus sequence, an initiation codon, either a 3xflag (in NT17, NT13) or a 3xHA (in NT15, see Methods S1 epitope site, a BstEII site (a 7-cutter, therefore asymmetric) for fluorescent protein insertion, and the coding sequence of hNKCC1. We used monomeric [4]YFP insensitive to Cl^−^ [5] (YFP/Q69M A206K, [6] ), monomeric Venus YFP with increased Cl- sensitivity (EYFP/V163S A206K, [6], [7]) and monomeric Cerulean CFP (ECFP/S72A Y145A H148D A206K, [8]), and mCherry as N-terminal fluorescent protein tags, with somewhat modified synthetic sequences to eliminate restriction sites and to add diagnostic sites and flanking BstEII sites. Of these, the YFP and CFP constructs were very well expressed, but the mCherry constructs generally showed a weak punctate fluorescence pattern and were not further investigated.

The new hNKCC1 cDNA was synthesized by Bio Basic (Toronto, Canada) in 5 pieces in pUC19 with no additional cloning sites, and subcloned in several steps into pcDNA3.1-neo. Other synthesized cDNAs included the alternate N-terminal tag and the several fluorescent protein sequences from Bio Basic and GenScript (Piscataway, NJ). The full length sequence was moved between vectors including pcDNA3.1 hygro and zeo and modified puro-Ires [9] using the NheI and PmeI sites. Constructs and sequence information are available through Addgene, and sequence information is in the Methods S1. We retain our internal DNA ID’s for reference (NT1 to NT999); Table 1 (and Table 2, below) list the constructs described here..

**Table 1:** NKCC1 constructs.

Name	Mutation	Tag1	Tag2	Vector
HA-hNKCC1	native hNKCC1, 3 silent mutations (ref. 9)	HA		pJB
NT17	hNKCC1, 262 silent mutations	3xFlag	YFP(mVenus)	pcDNA3.1-neo
NT13	hNKCC1, 262 silent mutations	3xFlag	Cl-sensitive YFP (EYFP/V163S A206K)	pcDNA3.1-neo
NT15-H	hNKCC1, 262 silent mutations	3xHA	CFP(mCerulean)	pcDNA3.1-hygro
NT51	S170Q,T177A,S183R,S193E,T203A,T205A, T207Q,T212A,T217A,T230A,S242N,T266A, T268Q,T274E,T276R in NT17	3xFlag	YFP(mVenus)	pcDNA3.1-neo
NT931	2xHA epitope in ECL2 insert at H398 in NT17	3xFlag	YFP(mVenus)	pcDNA3.1-neo
NT933	2xHA epitope in ECL3 insert at K460 in NT17	3xFlag	YFP(mVenus)	pcDNA3.1-neo
NT935	2xHA epitope in ECL4 insert at F574 in NT17	3xFlag	YFP(mVenus)	pcDNA3.1-neo
Cys mutants	Cysteine mutants in NT17 -- see Table 2			

Residue positions refer to hNKCC1 sequence.

**Table 2:** Activity of NKCC1 cysteine mutants.

	Mutation								activity relative to NT17 (%)		
ID#	TM1	TM7	TM8	TM11	ECL4	CtA	CtB	CtC		err.	n
NT104	A								156	28	2
NT103	S								97	-	1
NT360		M							143	12	2
NT399			A						87	21	2
NT400			S						106	18	2
NT501				S,V					41	4	3
NT859	A	M	S	S,V					32	8	3
NT365					S,S,S,S				80	4	3
NT366					delete				54	6	3
NT572				S,V		S			178	19	2
NT573				S,V			S		152	13	2
NT674				S,V				S	146	7	2
NT575				S,V		S	S		129	18	2
NT862		M			S,S,S,S				25	3	2
NT863		M			delete				24	1	2
NT864				S,V	S,S,S,S				55	7	5
NT865				S,V	delete				55	6	5
NT866	A	M	S	S,V	S,S,S,S				25	3	2
NT867	A	M	S	S,V	delete				26	3	2
NT858	A	M	S	S,V		S	S	S	57	5	3
NT868	A	M	S	S,V	S,S,S,S	S	S	S	32	7	2
NT869	A	M	S	S,V	delete	S	S	S	27	2	2
HEK									18	3	6

Cysteine in the NT17 NKCC1 construct was replaced with alanine, methionine, or serine, as indicated in columns 2-9. Activity in 86Rb influx is expressed as a percent of the control flux in NT17-transfected cells; SEM or range of duplicates is indicated.

### Mutagenesis by synthesis and subcloning

A great advantage of the hNKCC1 cassette cDNA (fig. 1) is that mutations of arbitrary complexity are easily created by cDNA synthesis followed by one-step subcloning of the appropriate cassette fragment. At current rates, and assuming the mutated cassette is ordered as part of a larger cDNA to meet minimum purchase requirements, this costs approximately $50 per mutation, and uses only a single subcloning step to complete the mutated construct. The cassette hNKCC1 is of even greater value when combining mutations from different regions in the protein, since straightforward subcloning strategies are available and immediately obvious.

### cDNA sub-cloning using an in-gel ligation protocol for small fragments

The hNKCC1 cassette supports a one-step subcloning approach, involving the insertion of small cDNA fragments (<200 bp) into the full length construct. We have found the in-gel ligation method to be highly efficient even for blunt-end ligation of small fragments, and suited to simultaneous preparation of 20–60 constructs using small volumes and mutichannel pipette operation.. The details are given in Methods S1; the methods are adapted from “Easy Subcloning” by Michael Koelle (http://medicine.yale.edu/labs/koelle/www/Protocols_files/protocol_subcloning.html).

Final cDNAs were sequenced in one direction through both insertion sites.

### Functional expression of human NKCC1 from a synthetic gene

cDNAs in HEK-293 cells, functional assay with ^86^Rb influx, and measurement of fluorescent protein tags have been described in detail elsewhere [6], [9], [10]. In this study, we used “mixed stable” cell lines which have been selected in the appropriate antibiotic (here geneticin), but are not clonal. In some cases we select individual colonies from either the original tranfection or replating of the mixed stable line in order to isolate stable clonal cell lines.

Our fluorescence measurements of Cl^−^- sensitive YFP are carried out in a low-noise LED fluorometer designed for bottom-illumination of cover slips or 96-well plates [6]. In this study we adapted the fluorometer for robotic control of well and solution selection, and we use a continuous perfusion system consisting of inflow and outflow hypodermic tubing which intrudes 6.5 mm into the top of the well and a dual-channel peristaltic pump (5ml/min). As previously reported, control experiments in which the cell membrane is permeabilized with saponin have demonstrated that the fluorescence quenching in response to changes in [Cl^−^] is well-fit by a single Cl^−^ binding site on YFP [6]. Numerous control experiments have shown that when the cells have been pre-exposed to 0 Cl^−^ medium for 1 hr and then treated with saponin in 0 Cl- solution, there is no significant change in fluorescence, i.e. the fluorescence is at f_max_. Therefore in experiments reported here we calculate [Cl^−^]_­I_ from fluorescence using a Cl^−^-sensor K_dCl_ =  60 mM and f_max_ determined for a sample after 60 min in 0 Cl^−^ [6].

### Immunofluorescence

Transfected HEK Cells were grown on polylysine-coated coverslips, fixed with methanol for 5 min, washed with PBS, and incubated in 0.1% bovine serum albumin (BSA) in phosphate-buffered saline (PBS) for 30 min at room temperature followed by incubations in either anti-HA (monoclonal, Covance Research products) or anti-FLAG (polyclonal, Sigma Aldrich) antibody (1∶500 in 0.1% BSA in PBS overnight at 4°C and followed by either anti-mouse or anti-rabbit Alexa-488 (Life Technologies) secondary for 1 hour (room temperature). Cells were subsequently incubated in TO-PRO-3 iodide (Life Technologies) for 15 min and then washed and mounted with Vectashield (Vector Laboratories). For extracellular epitope labeling, cells were washed with ice cold PBS and incubated in 0.1% BSA for 30 min at 4°C, followed by incubation in anti-HA antibody overnight washed and anti-mouse Alexa-568 secondary for 4 hours. Cells were then washed with PBS and fixed with methanol for 5 min followed by incubation with Anti-FLAG antibody as described above. Images were obtained using a laser scanning confocal microscope (Zeiss LSM 710; Carl Zeiss).

## Results and Discussion

### Functional expression of human NKCC1 from a synthetic cDNA

**Figure 2:**
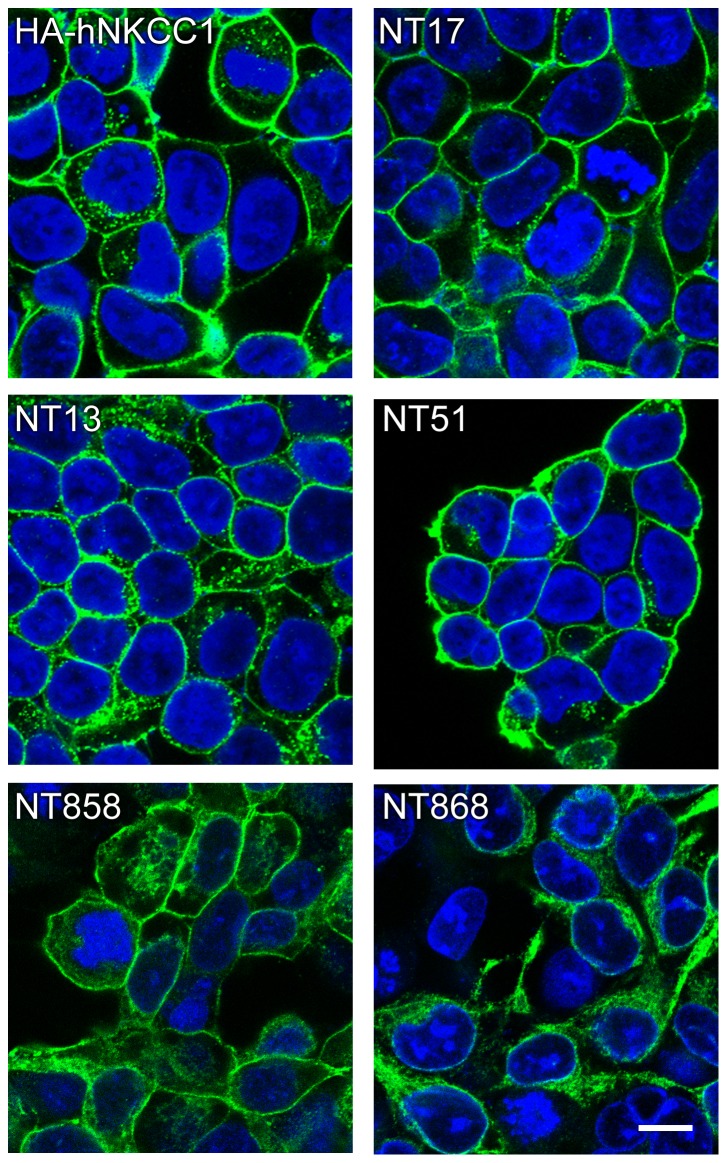
Confocal immunofluorescence images of NKCC1 cell lines. After fixation, NKCC1 is visualized using anti-HA-antibody (HA-hNKCC1) or anti FLAG-antbody (green, Alexa 488 secondary). Nuclei are stained with TO-PRO-3-iodide (Life Technologies). Shown are HA-tagged hNKCC1 [9], 3xFlag-YFP-tagged hNKCC1 (NT17), 3xFlag-Y’FP-tagged hNKCC1 (NT13), NT17 with threonines and serines mutated in the N-terminus phosphoregulatory domain (NT51), TM and intracellular cysless NKCC1 (NT858), and cysless NKCC1 (NT868). The marker in the lower right corner is 10 µm.

Expresion of hNKCC1 from the new synthetic cDNA construct (fig. 1A, above) was readily achieved in HEK cells. As illustrated in fig. 2, the new constructs tagged with 3XFlag epitope and YFP (NT17,NT13) are appropriately trafficked to the cell membrane with high efficiency, similarly to HA-tagged hNKCC1 expressed from the native cDNA. In contrast, NT17 with all cysteines removed by mutation is largely restricted to the endoplasmic reticulum (NT868, bottom right) – this and other constructs will be discussed below.

**Figure 3:**
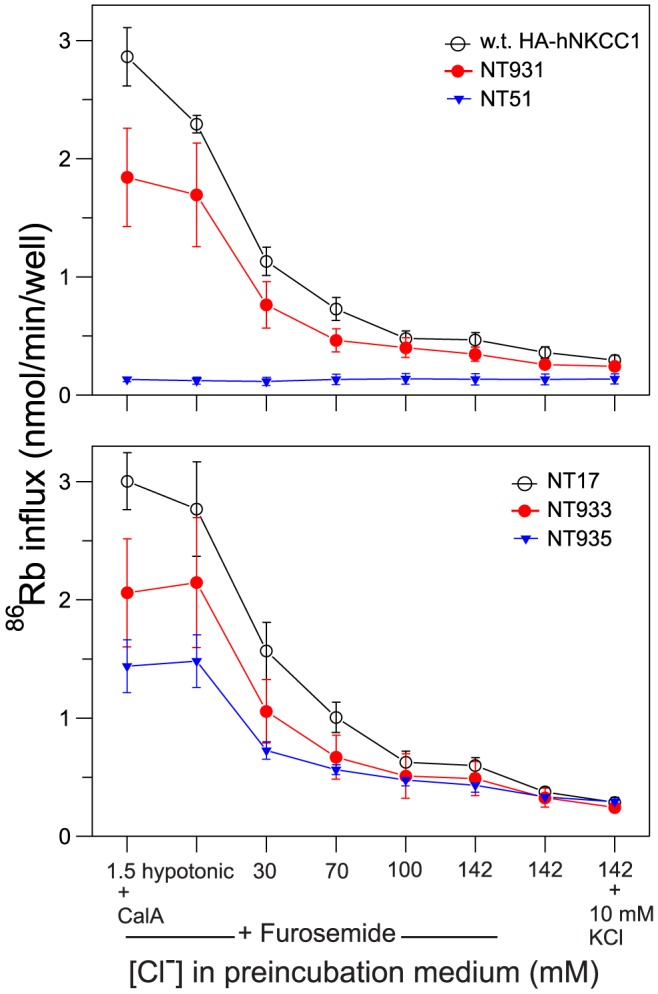
Activity of NKCC1 constructs upon activation in low Cl^−^ media. ^86^Rb influx was determined in a 1-min 96-well plate influx assay after 45 min of preincubation of cells in various media as indicated. Data are shown for two constructs with wild-type hNKCC1 sequence (HA-hNKCC1, upper panel; NT17, lower panel), for hNKCC1 with threonines and serines mutated in the N-terminus phosphoregulatory domain (upper panel, NT51), and for three constructs with extracellular epitope tags (NT931, NT933, NT935). Furosemide is a rapidly reversible inhibitor included during pre-incubation to eliminate effects of NKCCs in that period, then removed prior to the flux. Calyculin-A (CalA) is a protein phosphatase-1 inhibitor added 15 min prior to the flux to achieve maximal phosphorylation and flux rate. Data are mean and S.E. from 3-6 experiments.

NKCC1 is activated by phosphorylation in its N-terminus in response to reduced intracellular [Cl^−^] [11]. As illustrated in fig. 3, the fluorescent protein tagged NKCCs have similar activation responses compared to the wild-type transporter indicating that the presence of the fluorescent protein tag spaced ∼200 amino acids from the phosphoregulatory domain has no significant effect on transporter function. This is fully consistent with previous findings with other NKCCs tagged at the N-terminus [12], [13].

**Figure 4:**
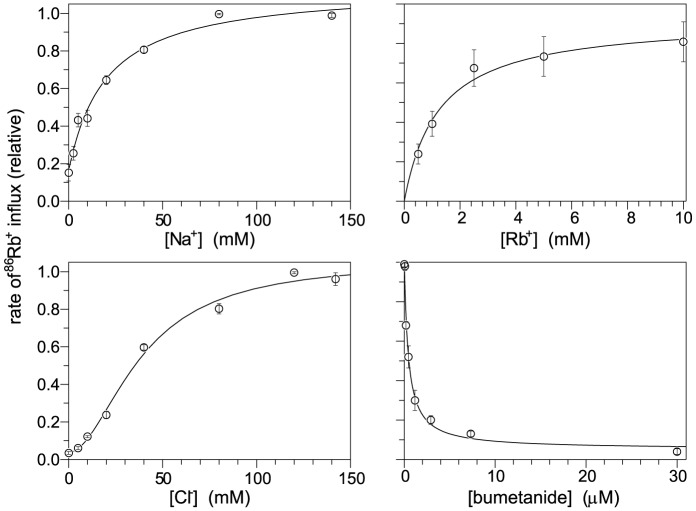
Activity of NKCC1 depends on Na^+^, Rb^+^, Cl^−^ and bumetanide concentration. ^86^Rb influx was determined in HEK cells expressing 3xflag-YFP-tagged hNKCC1 (NT17). Data points are relative to the maximum flux and are the average from three experiments with SEM. Lines are the best fit to a single binding site or in the case of [Cl^−^] dependence, to the hill equation (n = 1.8). The K_m_ values are (averages of determinations in three experiments): K_m(Na)_ 20.0±2.7 mM, K_m(Rb)_ 1.523±0.40 mM, K_m(Cl)_ 42.8±4.1 mM, and K_i (bumet)_ 0.46±0.10 µM; these are essentially the same as previously determined for the HA-tagged wild type human NKCC1 [21]: K_m(Na)_ 18.1±1.6 mM, K_m(Rb)_ 1.65±0.16 mM, K_m(Cl)_ 47.9±1.6 mM, and K_i (bumet)_ 0.42±0.02 µM.

NKCCs require Na^+^, K^+^ (or Rb^+^) and Cl^−^ for net transport to occur, as illustrated in fig. 4 for NT17. The N-terminal fluorescent protein does not significantly affect the quantitative transport behavior as seen from comparison of NT17 with native NKCC1 (fig. 4, see K_m_ values in legend). While fully expected, this is the first time this observation has been made.

### A phospho-dead NKCC1 mutant has no transport activity

We have previously shown that activation of NKCC1 requires the phosphorylation of two threonines in the N-terminus [10], but it is clear that at least 6 other residues in the same sequence region can become phosphorylated [10], [14]. In NKCC2, it seems that this larger group of sites must become phosphorylated in order to bring about maximum activation [15]. As a completely “phospho-dead” construct, useful in studies of the NKCC1 activation mechanism, we mutated 11 threonines and 4 serines in this phosphoregulatory region. This construct (NT51) was expressed in HEK cells and was effectively delivered to the plasma membrane (fig. 2), but without the appropriate phosphoacceptor sites it was functionally inactive under all conditions (fig. 3, top).

### NKCC1 function determined with a Cl-sensing YFP tag

The activity of Na-K-Cl cotransporters has generally been determined by ^86^Rb^+^ influx measurements, relying on the fact that Rb^+^ is transported almost the same as K^+^. This method has the advantage of good signal-noise, since in most cells non-NKCC mediated Rb^+^ influxes are very small after inhibiting Na-pumps with ouabain. The method can however be seen as inconvenient because of the radioactivity involved, and may be somewhat cumbersome to adapt to high-throughput technology. Several fluorometric approaches to assay the function of NKCC have been used, including sensing intracellular Cl^−^ with MQAE dye [16], sensing NKCC-mediated NH4+ influx with a pH sensing dye [17], and sensing NKCC-mediated Tl+ influx with FluxOR dye [18]. Each of these methods has its disadvantages in terms of sensitivity, dynamic range, and faithfulness in mimicking the canonical Na^+^+K^+^+Cl^−^ cotransport.

**Figure 5:**
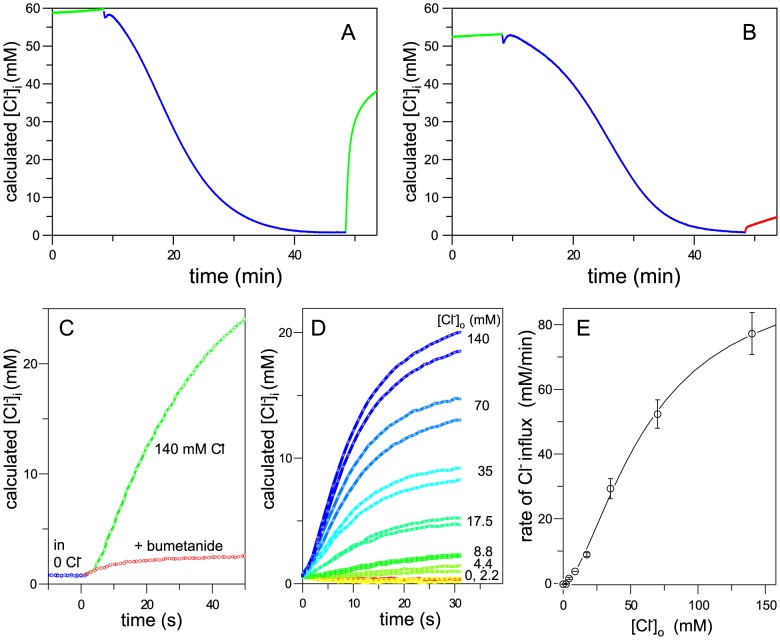
NKCC1 activity determined with Cl^−^-sensing YFP. NT13 cells on cover slips (a,b,c) or in a 96-well plate (d,e) were perfused with various media and intracellular Cl^−^ concentration was calculated from YFP fluorescence as described in Methods. A,B,C. Cells were perfused with regular medium (green), 0 Cl^−^ medium (blue), or regular medium with 250 µM bumetanide (red) as shown. Traces from the two coverslips studied in A and B are replotted in C on an expanded time scale starting at ∼48 min. D. Similar to C except cells are in individual wells of a 96-well plate and are monitored on return to media of different [Cl^−^]. E. Rate of Cl^−^ influx as a function of [Cl^−^]_ext_, evaluated as the slope of the data in (D) during the linear phase. The least-squares fit of the data to the Hill equation is shown (K_m_ = 62 mM, n = 1.62).

We have previously reported that YFP engineered to have moderately high sensitivity to Cl­^−^ [7] gives a convenient readout of intracellular [Cl^−^] in NKCC-transfected HEK cells [6]. We thus included this probe in place of the N-terminal YFP tag in NT17 and expressed the construct (NT13) in HEK cells. As illustrated in fig. 5a, a slow decrease in intracellular Cl^−^ occurs with prolonged incubation of cells in 0 Cl^−^ medium, and a very rapid influx of Cl^−^ occurs when Cl^−^ is added back to the medium. The rate of this influx is dramatically blunted by bumetanide (fig. 5b) demonstrating that Cl^−^ regain is NKCC-mediated – with the initial rate expanded in fig. 5c, it is seen that bumetanide inhibits >95% of the flux.

The Cl^−^-sensing YFP-NKCC is readily adapted to high throughput methods – the data shown in fig. 5d were obtained at different extracellular Cl^−^ concentrations in different wells of a 96-well plate under robotic control – as seen, the method is highly sensitive and reproducible. The dependence of Cl^−^ influx as a function of [Cl^−^]_ext_ is shown in fig. 5e and is very similar to the dependence of ^86^Rb^+^ influx on [Cl^−^]_ext_ in fig. 4. This assay has the advantage that it utilizes a well-defined state of the Na-K-Cl cotransporter – after 0 Cl^−^ preincubation, the transporter is fully activated [10], and the consequent Cl- influx is a net flux of Cl^−^ in a “zero-trans” situation. It may be noted that the Cl^−^-sensor is included as a tag on NKCC simply as a convenience - the assay would work as well with a Cl^−^- sensing YFP expressed on its own.

### Extracellular epitope tags to detect NKCC1 at the cell surface

A marker of NKCC available at the outside of the membrane would facilitate many types of studies including live cell sorting of cells based on NKCC, studies of membrane trafficking of the protein, and other studies involving identification of the fraction of NKCC localized to the plasma membrane. Previous efforts with NKCCs have employed surface biotinylation, but there are many limitations of this chemical method. We have sought for some time to engineer an antibody epitope into the extracellular loops of NKCCs by modification of the existing protein sequence to that of HA or flag epitopes – these efforts have always lead to constructs which are not effectively delivered to the plasma membrane (unpublished observations).

**Figure 6:**
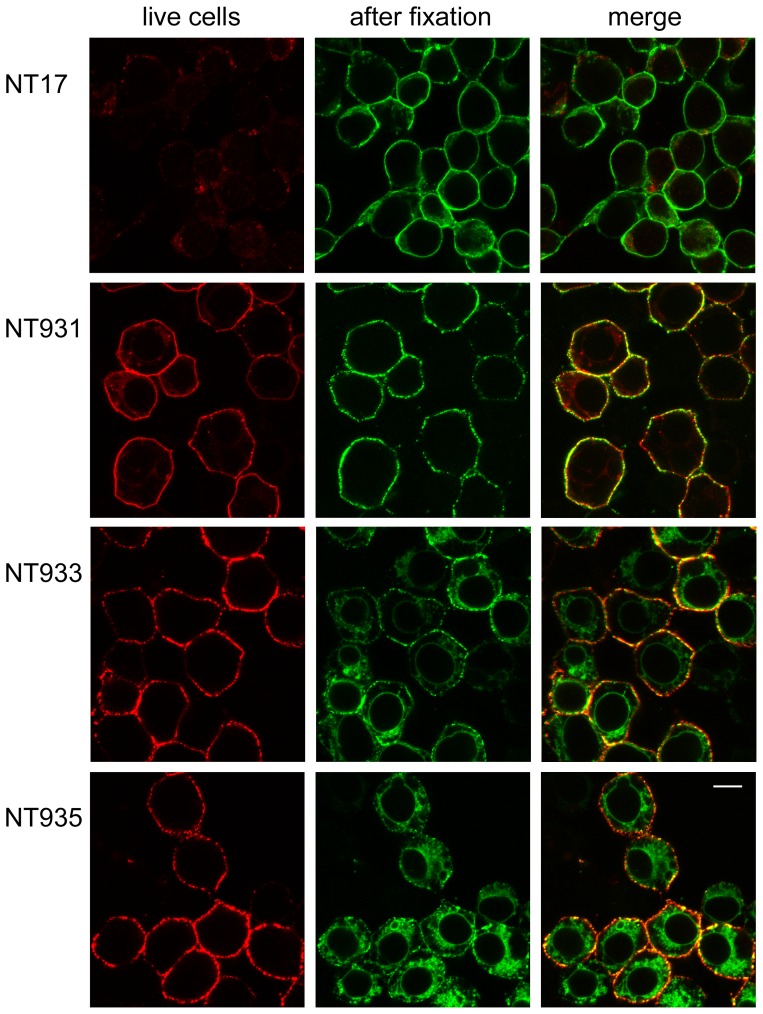
Extracellular epitope tags for NKCC1. NT17 and the three extracellular HA-epitope-tagged constructs were grown on polylysine-coated coverslips, and sequentially exposed with appropriate PBS-BSA washes, to HA-antibody overnight at 4°C (red channel), Alexa 568 anti-mouse secondary, methanol as fixative for 5 min, then Flag-antibody (green channel), and Alexa 488 anti-rabbit secondary. The scale bar in the bottom right panel represents 10 µm.

Here we attempted simple insertions of 2xHA-epitope sequence, retaining all native amino acids – insertion points (blue diamonds in fig. 1B) were chosen from gap points in predicted extracellular regions in multiple sequence alignments of NKCCs and KCCs. As shown in the left column of fig. 6, each of these constructs is very well labeled by HA-antibody applied to the intact cell, and ^86^Rb influx studies confirm that they are functional in the membrane (NT931, NT933, NT935, fig. 2). In contrast, control NT17 cells, in which the extracellular epitope is absent, are not labeled with antibody under similar conditions (fig. 6 top). After fixation, NKCC is identified in all cells (green in fig. 6), and it is clear that there is a substantial amount of intracellular NKCC that becomes exposed upon fixation. This is especially true of the ECL3 and ECL4 tags (NT933 and NT935) which appear to be less well delivered to the membrane compared to the wild type NKCC1, and would therefore be second-choice candidates for experiments requiring that a large fraction of NKCC1 is at the membrane. On the other hand, the majority of the ECL2 epitope tag in NT931 is labeled in intact cells, showing that this transporter is very effectively processed and delivered to the plasma membrane.

### Towards a “cys-less” hNKCC1

Because of the unique chemical reactivity of cysteine, mutation of native amino acids to cysteine has proven to be a powerful tool in many types of structure-function studies of membrane proteins. Cysteine mutagenesis is the starting point for studies involving site-specific covalent labeling with fluorescent tags, for cysteine-scanning accessibility studies of membrane proteins [19], and for site-specific disulfide-mediated crosslinking of proteins [20]. To carry out such experiments without interference from native cysteines, it is desirable if not necessary to remove all of the native cysteines from the protein. Here we report our partial success with NKCC1.

There are 12 native cysteines in hNKCC1. Five are predicted in the transmembrane domain, one each in TMs 1, 7 and 8, and two together in TM11. As tabulated in the first four lines of Table 1, mutagenesis of each of these to serine, alanine, or methionine resulted in transporters that were trafficked to the membrane and exhibited at least 75% of the flux of the wild type NT17 transporter (table 2). Removal of all 5 TM cysteines yielded a transporter (NT859) with substantially reduced activity that would be problematic for general use, but sufficiently active for appropriate control experiments; paradoxically the observed activity was greater when cytoplasmic cysteines were also mutated (NT858, table 2 and fig. 2).

We expect that the four extracellular cysteines in ECL4 are paired natively as disulfides and are unlikely to interfere with most cysteine chemistry – in fact we have not seen interference from these cysteines in labeling experiments with MTS and maleimide reagents (unpublished). Nonetheless we removed these residues together either by mutagenesis to serine or by deletion of much of the ECL4 loop. As reported in table 2, approximately 80% or 54% of the activity was retained, respectively. The three intracellular cysteines appear less important – we found little significant change in activity removing one or all of these (table 2).

The deleterious effects of cysteine removal on NKCC trafficking became more pronounced in a non-linear fashion when we combined TM cysteine removal with either extracellular or intracellular cysteine removal, or both. As illustrated by the fully cys-less construct NT868 in fig. 2, these mutants were retained within the endoplasmic reticulum and had no detectable NKCC activity above background (table 2). It is of course possible that greater success could be achieved with other amino acid substitutions, but this is not possible to predict.

In conclusion, we have reported a sensitive new NKCC assay method in addition to important advances in extracellular epitopes and cysteine removal in the human NKCC1 protein. These findings are all the more useful in the context of the synthetic cDNA encoding NKCC1, because numerous convenient restriction sites guarantee easy combining of these features with new mutations and in different combinations. It is anticipated that the advances and constructs reported here will be of enormous value in the ongoing study of NKCCs.

## Supporting Information

Methods S1 **A. cDNA sub-cloning.using an in-gel ligation protocol for small fragments. B. cDNA sequences**.
(DOC)
