# Supplementary material for: Functional Expression of Human NKCC1 from a Synthetic Cassette-Based cDNA: Introduction of Extracellular Epitope Tags and Removal of Cysteines
Source: PLoS One. 2013 Dec 5;8(12):e82060. doi: 10.1371/journal.pone.0082060 (PMC3855340; doi:10.1371/journal.pone.0082060)
Supplement: Methods S1 — A. cDNA sub-cloning.using an in-gel ligation protocol for small fragments. B. cDNA sequences. (DOC) [file pone.0082060.s001.doc]

Methods S1.

A. cDNA sub-cloning.using an in-gel ligation protocol for small fragments.

The hNKCC1 cassette supports a one-step subcloning approach, involving the insertion of small cDNA fragments (<200 bp) into the full length construct. We have found the in-gel ligation method to be highly efficient even for blunt-end ligation of small fragments, and suited to simultaneous preparation of 20-60 constructs. The method is modified for small volumes and mutichannel pipette operation from “Easy Subcloning” by Michael Koelle (<http://medicine.yale.edu/labs/koelle/www/Protocols_files/protocol_subcloning.html>). All operations including bacterial transformation are carried out in PCR strip tubes using multichannel pipettes. These methods are 80-100% successful, with failures traced primarily to low DNA in the starting insert, inaccurate excision of gel bands, and to pipetting errors.

DNA was digested in 20 ul volume containing 1ul each restriction enzyme (New England Biolabs), 2 ul 10x buffer with BSA, and cDNA (approx 3 ug for small fragments, approx 0.2 ug for vectors) and 2 hr incubation at appropriate temperature. All vector constructs were treated with intestinal phosphatase (rAPid, Roche Diagnostics) for 30 min at 37^o^C at the end of the incubation. cDNA was run on 0.8% SeaPlaque GTG low melt agarose or 2% NuSieve GTG (for fragments <300 bp) and visualized with ethidium bromide. A CCD camera photograph of the gel with enhanced contrast was printed at exactly full size to use as a cutting guide (using bubbles or wire markers for exact alignment), and aligned to the gel in a plastic envelope. A flattened and sharpened piece of brass tubing (0.14” o.d.) was used to excise 30-40 ul gel per band. In this way many bands could be carefully excised on a lightbox with white light – correct excision was confirmed by rephotographing the gel after all bands were excised.

In-gel ligation. In-gel ligation reactions were carried out in a 10 ul volume, including 4 ul of gel containing the insert, 2 ul of gel containing the vector, 1 ul T4 ligase (New England Biolabs), and 1 ul 10x buffer. In practice, gel slices (in strip tubes) were melted at 72^o^C for 2 min in a PCR machine and cooled to 40^o^C holding temperature, under which condition gel was pipetted and mixed into ligation strip tubes also at 40^o^C. After cooling these tubes for a few seconds, the T4 mix (4ul,0^o^C) was added and mixed using a multichannel pipette and the ligation tubes placed on ice. Ligation proceeded for 8-24 hr at 15^o^C or 2 hr at room temperature.

Transformation. In-gel ligation reactions were melted at 72^o^C for 2 min, 40 ul 0.1 M Tris pH 7.4 was added and mixed, and samples placed on ice for 3-10 min. 20 ul of the mixture was added to 20 ul competent DH5a cells in another strip tube at 0^o^C. After 30 min at 0^o^C and 50s heat shock at 42^o^C, addition of 150 ul LB medium and additional 1 hr incubation at 37^o^C, cells were plated on AMP plates. The background is so low with phosphatase-treated vectors and small inserts that we seldom use control transformations.

B. cDNA sequences.

>NT17 3X-Flag-YFP(mVenus)-tagged human NKCC1. Synthetic DNA, convenient sites, in pcDNA3.1-neo.

GCTAGCCCGGGCACGGCCGCCACCATGGGAGACTACAAAGACCATGACGGTGATTATAAAGATCACGACATCGACTACAA

GGATGACGATGACAAGGGTGACCCTACCGTCAGCAAGGGCGAGGAGCTGTTCACCGGGGTGGTGCCCATCCTGGTCGAGC

TGGACGGCGACGTAAACGGCCACAAGTTCAGCGTGTCCGGCGAGGGCGAGGGCGATGCCACCTACGGCAAGCTGACCCTG

AAGTTCATCTGCACCACCGGCAAGCTGCCCGTGCCCTGGCCCACCCTCGTGACCACCTTCGGCTACGGCCTGATGTGCTT

CGCCCGCTACCCCGACCACATGAAGCAGCACGACTTCTTCAAGTCCGCCATGCCCGAAGGCTACGTCCAGGAGCGCACCA

TCTTCTTCAAGGACGACGGCAACTACAAGACCCGCGCCGAGGTGAAGTTCGAGGGCGACACCCTGGTGAACCGCATCGAG

CTGAAGGGCATCGACTTCAAGGAGGACGGCAACATCCTAGGGCACAAGCTGGAGTACAACTACAACAGCCACAACGTTTA

TATCATGGCCGACAAGCAGAAGAACGGCATCAAGGTGAACTTCAAGATCCGCCACAACATCGAGGACGGCAGCGTGCAGC

TCGCCGACCACTACCAGCAGAACACCCCCATCGGCGACGGCCCCGTGCTGCTGCCCGACAACCACTACCTGAGCTACCAG

TCCAAACTGAGCAAAGACCCCAACGAGAAGCGCGATCACATGGTCCTGCTGGAGTTCGTGACCGCCGCCGGGATCACTCT

CGGCATGGACGAGCTCTACAAGGGTGACCTGGAGCCGAGGCCCACGGCTCCTAGCTCCGGCGCGCCAGGCCTCGCCGGAG

TCGGCGAAACGCCGAGCGCCGCTGCGCTGGCCGCAGCCAGGGTGGAACTGCCCGGAACCGCTGTGCCCTCGGTGCCGGAG

GATGCTGCGCCCGCGAGCCGGGACGGGGGCGGGGTCCGCGATGAGGGACCAGCCGCCGCCGGTGACGGCCTGGGCAGACC

CTTGGGGCCTACCCCGAGCCAGAGCCGTTTCCAGGTGGACCTCGTTTCCGAGAACGCAGGGCGGGCTGCTGCTGCGGCGG

CGGCGGCTGCTGCGGCCGCCGCCGCTGCTGGTGCCGGGGCGGGGGCCAAGCAGACTCCTGCCGACGGGGAAGCCAGCGGC

GAGAGCGAGCCAGCTAAAGGCAGCGAGGAAGCCAAAGGCCGCTTCCGCGTTAACTTCGTAGATCCTGCTGCCAGCTCGTC

CGCAGAAGATAGCCTGTCAGATGCCGCCGGGGTCGGAGTCGATGGACCCAACGTGAGCTTCCAGAACGGCGGGGACACGG

TACTGAGCGAGGGCAGCAGCCTCCATTCCGGTGGCGGCGGCGGCTCCGGCCACCACCAGCACTACTATTATGATACCCAC

ACCAACACCTACTATCTGCGCACCTTCGGCCACAACACAATGGACGCGGTACCCAGGATCGACCACTACCGGCACACAGC

CGCGCAGCTCGGCGAGAAGCTGCTCCGGCCTAGCCTGGCCGAGCTGCATGACGAGCTGGAAAAGGAACCTTTTGAGGATG

GCTTTGCAAATGGGGAAGAAAGCACTCCAACACGTGATGCTGTGGTCACGTACACGGCCGAAAGTAAAGGAGTCGTGAAG

TTTGGCTGGATCAAGGGTGTACTAGTACGTTGTATGTTAAACATTTGGGGTGTGATGCTTTTCATTCGTCTCTCATGGAT

TGTGGGACAAGCTGGAATTGGTCTATCAGTCCTTGTAATAATGATGGCTACTGTTGTGACAACTATCACAGGATTGTCCA

CTAGCGCCATCGCAACTAATGGATTTGTAAGGGGAGGCGGCGCATATTATTTAATATCTAGAAGTCTAGGGCCTGAATTT

GGTGGTGCCATTGGTCTAATCTTCGCCTTTGCCAACGCTGTTGCTGTTGCTATGTATGTGGTTGGATTTGCAGAAACCGT

GGTGGAGTTGCTGAAAGAACATTCCATTCTTATGATAGATGAAATCAATGACATCCGGATTATtGGAGCCATTACAGTCG

TGATTCTTTTAGGTATCTCAGTAGCCGGTATGGAGTGGGAAGCAAAAGCTCAGATTGTTCTTTTGGTGATCCTACTTCTT

GCTATTGGTGATTTCGTCATAGGAACATTTATCCCACTCGAGAGCAAGAAGCCAAAAGGGTTTTTTGGCTATAAATCTGA

AATCTTTAATGAGAACTTTGGCCCCGATTTTCGAGAAGAAGAAACTTTCTTTTCTGTATTTGCCATCTTTTTTCCTGCTG

CAACTGGAATTCTGGCTGGCGCAAATATCTCAGGTGATCTTGCAGATCCTCAGTCAGCCATACCCAAAGGAACACTCCTA

GCCATTTTAATTACTACATTGGTTTATGTAGGAATTGCCGTGTCTGTAGGTTCTTGTGTCGTACGCGATGCCACTGGTaA

TGTTAATGACACTATCGTAACAGAGCTAACAAACTGTACTAGCGCAGCCTGCAAATTAAACTTTGATTTTTCATCTTGTG

AATCCAGTCCTTGTAGCTACGGCCTAATGAACAACTTTCAGGTAATGAGTATGGTGTCAGGATTTACACCACTAATTTCC

GCGGGCATATTCTCTGCCACTCTTTCTAGCGCATTAGCATCCCTAGTGAGTGCTCCCAAGATATTtCAGGCCCTATGTAA

AGACAACATCTACCCCGCTTTCCAGATGTTTGCTAAAGGTTATGGGAAAAATAATGAGCCATTACGTGGCTACATCTTAA

CATTCTTAATTGCACTTGGATTCATCTTAATTGCCGAACTGAATGTTATTGCACCAATTATCTCAAACTTCTTCCTTGCC

AGCTATGCCCTCATCAATTTTTCAGTATTCCATGCTTCACTGGCAAAAAGCCCAGGATGGCGACCGGCATTTAAATACTA

CAATATGTGGATCTCACTTCTTGGAGCAATTCTTTGTTGCATAGTAATGTTCGTCATTAACTGGTGGGCTGCTTTGCTAA

CCTACGTGATAGTCCTTGGGCTGTATATTTATGTTACCTACAAAAAACCCGATGTGAATTGGGGATCCTCTACACAAGCC

CTGACTTACCTCAATGCCCTGCAACATAGTATTAGGCTTTCTGGCGTGGAGGACCATGTGAAAAACTTTAGGCCACAGTG

TCTTGTTATGACAGGAGCGCCAAACTCACGGCCGGCCTTACTTCATCTTGTTCACGATTTCACAAAAAATGTTGGTTTGA

TGATCTGTGGACACGTACACATGGGACCACGCAGACAAGCCATGAAAGAAATGTCCATCGACCAAGCCAAATATCAGCGC

TGGCTTATTAAGAACAAAATGAAGGCTTTTTATGCACCAGTACATGCAGATGACCTCAGAGAAGGGGCCCAATACTTGAT

GCAGGCTGCTGGCCTGGGCCGTATGAAGCCAAACACACTAGTCCTTGGATTTAAGAAAGATTGGTTGCAAGCAGATATGA

GGGATGTGGATATGTACATAAACTTATTTCACGATGCTTTTGACATACAATATGGCGTAGTGGTTATTCGCCTAAAAGAA

GGTCTGGATATTAGTCACCTGCAGGGACAAGAAGAATTATTGTCATCACAAGAGAAATCTCCTGGCACCAAGGATGTCGT

AGTAAGTGTGGAATATAGTAAAAAGTCCGATTTAGATACTTCGAAACCCCTCAGTGAAAAACCAATTACCCATAAAGTCG

AGGAAGAAGATGGCAAGACTGCAACTCAACCACTGTTGAAAAAAGAATCCAAAGGCCCTATTGTGCCTTTGAATGTAGCT

GACCAAAAGCTTCTTGAAGCTAGTACACAGTTTCAGAAAAAACAAGGAAAGAATACTATTGATGTCTGGTGGCTTTTTGA

TGACGGAGGTCTGACCTTATTGATACCTTACCTTCTGACGACCAAGAAAAAATGGAAAGACTGTAAGATCAGAGTATTCA

TTGGTGGAAAGATAAACAGAATAGACCATGACCGGAGAGCGATGGCTACTTTACTGAGCAAGTTCAGAATCGATTTTTCT

GACATCATGGTTCTAGGAGACATCAATACCAAACCAAAGAAAGAAAACATTATAGCTTTCGAGGAAATCATTGAGCCATA

CAGACTTCACGAAGATGATAAAGAGCAAGATATCGCAGATAAAATGAAAGAAGATGAACCCTGGCGAATAACAGATAATG

AATTGGAGCTGTATAAGACCAAGACATACCGGCAGATCAGGTTAAATGAGTTATTAAAGGAACATTCAAGCACAGCTAAC

ATTATTGTGATGAGTTTACCGGTCGCACGAAAGGGTGCCGTTTCTAGTGCTCTCTACATGGCATGGTTAGAAGCACTATC

TAAGGATCTACCACCAATCCTACTAGTTCGTGGGAATCATCAGAGTGTCCTTACCTTCTATTCATAATTAATTAAGTTTA

AACCCGCTGATCAGCCTCGACTGTGCCTTCTAGTTGCCAGCCATCTGTTGTTTGCCCCTCCCCCGTGCCTTCCTTGACCC

TGGAAGGTGCCACTCCCACTGTCCTTTCCTAATAAAATGAGGAAATTGCATCGCATTGTCTGAGTAGGTGTCATTCTATT

CTGGGGGGTGGGGTGGGGCAGGACAGCAAGGGGGAGGATTGGGAAGACAATAGCAGGCATGCTGGGGATGCGGTGGGCTC

TATGGCTTCTGAGGCGGAAAGAACCAGCTGGGGCTCTAGGGGGTATCCCCACGCGCCCTGTAGCGGCGCATTAAGCGCGG

CGGGTGTGGTGGTTACGCGCAGCGTGACCGCTACACTTGCCAGCGCCCTAGCGCCCGCTCCTTTCGCTTTCTTCCCTTCC

TTTCTCGCCACGTTCGCCGGCTTTCCCCGTCAAGCTCTAAATCGGGGGCTCCCTTTAGGGTTCCGATTTAGTGCTTTACG

GCACCTCGACCCCAAAAAACTTGATTAGGGTGATGGTTCACGTAGTGGGCCATCGCCCTGATAGACGGTTTTTCGCCCTT

TGACGTTGGAGTCCACGTTCTTTAATAGTGGACTCTTGTTCCAAACTGGAACAACACTCAACCCTATCTCGGTCTATTCT

TTTGATTTATAAGGGATTTTGCCGATTTCGGCCTATTGGTTAAAAAATGAGCTGATTTAACAAAAATTTAACGCGAATTA

ATTCTGTGGAATGTGTGTCAGTTAGGGTGTGGAAAGTCCCCAGGCTCCCCAGCAGGCAGAAGTATGCAAAGCATGCATCT

CAATTAGTCAGCAACCAGGTGTGGAAAGTCCCCAGGCTCCCCAGCAGGCAGAAGTATGCAAAGCATGCATCTCAATTAGT

CAGCAACCATAGTCCCGCCCCTAACTCCGCCCATCCCGCCCCTAACTCCGCCCAGTTCCGCCCATTCTCCGCCCCATGGC

TGACTAATTTTTTTTATTTATGCAGAGGCCGAGGCCGCCTCTGCCTCTGAGCTATTCCAGAAGTAGTGAGGAGGCTTTTT

TGGAGGCCTAGGCTTTTGCAAAAAGCTCCCGGGAGCTTGTATATCCATTTTCGGATCTGATCAAGAGACAGGATGAGGAT

CGTTTCGCATGATTGAACAAGATGGATTGCACGCAGGTTCTCCGGCCGCTTGGGTGGAGAGGCTATTCGGCTATGACTGG

GCACAACAGACAATCGGCTGCTCTGATGCCGCCGTGTTCCGGCTGTCAGCGCAGGGGCGCCCGGTTCTTTTTGTCAAGAC

CGACCTGTCCGGTGCCCTGAATGAACTGCAGGACGAGGCAGCGCGGCTATCGTGGCTGGCCACGACGGGCGTTCCTTGCG

CAGCTGTGCTCGACGTTGTCACTGAAGCGGGAAGGGACTGGCTGCTATTGGGCGAAGTGCCGGGGCAGGATCTCCTGTCA

TCTCACCTTGCTCCTGCCGAGAAAGTATCCATCATGGCTGATGCAATGCGGCGGCTGCATACGCTTGATCCGGCTACCTG

CCCATTCGACCACCAAGCGAAACATCGCATCGAGCGAGCACGTACTCGGATGGAAGCCGGTCTTGTCGATCAGGATGATC

TGGACGAAGAGCATCAGGGGCTCGCGCCAGCCGAACTGTTCGCCAGGCTCAAGGCGCGCATGCCCGACGGCGAGGATCTC

GTCGTGACCCATGGCGATGCCTGCTTGCCGAATATCATGGTGGAAAATGGCCGCTTTTCTGGATTCATCGACTGTGGCCG

GCTGGGTGTGGCGGACCGCTATCAGGACATAGCGTTGGCTACCCGTGATATTGCTGAAGAGCTTGGCGGCGAATGGGCTG

ACCGCTTCCTCGTGCTTTACGGTATCGCCGCTCCCGATTCGCAGCGCATCGCCTTCTATCGCCTTCTTGACGAGTTCTTC

TGAGCGGGACTCTGGGGTTCGAAATGACCGACCAAGCGACGCCCAACCTGCCATCACGAGATTTCGATTCCACCGCCGCC

TTCTATGAAAGGTTGGGCTTCGGAATCGTTTTCCGGGACGCCGGCTGGATGATCCTCCAGCGCGGGGATCTCATGCTGGA

GTTCTTCGCCCACCCCAACTTGTTTATTGCAGCTTATAATGGTTACAAATAAAGCAATAGCATCACAAATTTCACAAATA

AAGCATTTTTTTCACTGCATTCTAGTTGTGGTTTGTCCAAACTCATCAATGTATCTTATCATGTCTGTATACCGTCGACC

TCTAGCTAGAGCTTGGCGTAATCATGGTCATAGCTGTTTCCTGTGTGAAATTGTTATCCGCTCACAATTCCACACAACAT

ACGAGCCGGAAGCATAAAGTGTAAAGCCTGGGGTGCCTAATGAGTGAGCTAACTCACATTAATTGCGTTGCGCTCACTGC

CCGCTTTCCAGTCGGGAAACCTGTCGTGCCAGCTGCATTAATGAATCGGCCAACGCGCGGGGAGAGGCGGTTTGCGTATT

GGGCGCTCTTCCGCTTCCTCGCTCACTGACTCGCTGCGCTCGGTCGTTCGGCTGCGGCGAGCGGTATCAGCTCACTCAAA

GGCGGTAATACGGTTATCCACAGAATCAGGGGATAACGCAGGAAAGAACATGTGAGCAAAAGGCCAGCAAAAGGCCAGGA

ACCGTAAAAAGGCCGCGTTGCTGGCGTTTTTCCATAGGCTCCGCCCCCCTGACGAGCATCACAAAAATCGACGCTCAAGT

CAGAGGTGGCGAAACCCGACAGGACTATAAAGATACCAGGCGTTTCCCCCTGGAAGCTCCCTCGTGCGCTCTCCTGTTCC

GACCCTGCCGCTTACCGGATACCTGTCCGCCTTTCTCCCTTCGGGAAGCGTGGCGCTTTCTCATAGCTCACGCTGTAGGT

ATCTCAGTTCGGTGTAGGTCGTTCGCTCCAAGCTGGGCTGTGTGCACGAACCCCCCGTTCAGCCCGACCGCTGCGCCTTA

TCCGGTAACTATCGTCTTGAGTCCAACCCGGTAAGACACGACTTATCGCCACTGGCAGCAGCCACTGGTAACAGGATTAG

CAGAGCGAGGTATGTAGGCGGTGCTACAGAGTTCTTGAAGTGGTGGCCTAACTACGGCTACACTAGAAGAACAGTATTTG

GTATCTGCGCTCTGCTGAAGCCAGTTACCTTCGGAAAAAGAGTTGGTAGCTCTTGATCCGGCAAACAAACCACCGCTGGT

AGCGGTTTTTTTGTTTGCAAGCAGCAGATTACGCGCAGAAAAAAAGGATCTCAAGAAGATCCTTTGATCTTTTCTACGGG

GTCTGACGCTCAGTGGAACGAAAACTCACGTTAAGGGATTTTGGTCATGAGATTATCAAAAAGGATCTTCACCTAGATCC

TTTTAAATTAAAAATGAAGTTTTAAATCAATCTAAAGTATATATGAGTAAACTTGGTCTGACAGTTACCAATGCTTAATC

AGTGAGGCACCTATCTCAGCGATCTGTCTATTTCGTTCATCCATAGTTGCCTGACTCCCCGTCGTGTAGATAACTACGAT

ACGGGAGGGCTTACCATCTGGCCCCAGTGCTGCAATGATACCGCGAGACCCACGCTCACCGGCTCCAGATTTATCAGCAA

TAAACCAGCCAGCCGGAAGGGCCGAGCGCAGAAGTGGTCCTGCAACTTTATCCGCCTCCATCCAGTCTATTAATTGTTGC

CGGGAAGCTAGAGTAAGTAGTTCGCCAGTTAATAGTTTGCGCAACGTTGTTGCCATTGCTACAGGCATCGTGGTGTCACG

CTCGTCGTTTGGTATGGCTTCATTCAGCTCCGGTTCCCAACGATCAAGGCGAGTTACATGATCCCCCATGTTGTGCAAAA

AAGCGGTTAGCTCCTTCGGTCCTCCGATCGTTGTCAGAAGTAAGTTGGCCGCAGTGTTATCACTCATGGTTATGGCAGCA

CTGCATAATTCTCTTACTGTCATGCCATCCGTAAGATGCTTTTCTGTGACTGGTGAGTACTCAACCAAGTCATTCTGAGA

ATAGTGTATGCGGCGACCGAGTTGCTCTTGCCCGGCGTCAATACGGGATAATACCGCGCCACATAGCAGAACTTTAAAAG

TGCTCATCATTGGAAAACGTTCTTCGGGGCGAAAACTCTCAAGGATCTTACCGCTGTTGAGATCCAGTTCGATGTAACCC

ACTCGTGCACCCAACTGATCTTCAGCATCTTTTACTTTCACCAGCGTTTCTGGGTGAGCAAAAACAGGAAGGCAAAATGC

CGCAAAAAAGGGAATAAGGGCGACACGGAAATGTTGAATACTCATACTCTTCCTTTTTCAATATTATTGAAGCATTTATC

AGGGTTATTGTCTCATGAGCGGATACATATTTGAATGTATTTAGAAAAATAAACAAATAGGGGTTCCGCGCACATTTCCC

CGAAAAGTGCCACCTGACGTCGACGGATCGGGAGATCTCCCGATCCCCTATGGTGCACTCTCAGTACAATCTGCTCTGAT

GCCGCATAGTTAAGCCAGTATCTGCTCCCTGCTTGTGTGTTGGAGGTCGCTGAGTAGTGCGCGAGCAAAATTTAAGCTAC

AACAAGGCAAGGCTTGACCGACAATTGCATGAAGAATCTGCTTAGGGTTAGGCGTTTTGCGCTGCTTCGCGATGTACGGG

CCAGATATACGCGTTGACATTGATTATTGACTAGTTATTAATAGTAATCAATTACGGGGTCATTAGTTCATAGCCCATAT

ATGGAGTTCCGCGTTACATAACTTACGGTAAATGGCCCGCCTGGCTGACCGCCCAACGACCCCCGCCCATTGACGTCAAT

AATGACGTATGTTCCCATAGTAACGCCAATAGGGACTTTCCATTGACGTCAATGGGTGGAGTATTTACGGTAAACTGCCC

ACTTGGCAGTACATCAAGTGTATCATATGCCAAGTACGCCCCCTATTGACGTCAATGACGGTAAATGGCCCGCCTGGCAT

TATGCCCAGTACATGACCTTATGGGACTTTCCTACTTGGCAGTACATCTACGTATTAGTCATCGCTATTACCATGGTGAT

GCGGTTTTGGCAGTACATCAATGGGCGTGGATAGCGGTTTGACTCACGGGGATTTCCAAGTCTCCACCCCATTGACGTCA

ATGGGAGTTTGTTTTGGCACCAAAATCAACGGGACTTTCCAAAATGTCGTAACAACTCCGCCCCATTGACGCAAATGGGC

GGTAGGCGTGTACGGTGGGAGGTCTATATAAGCAGAGCTCTCTGGCTAACTAGAGAACCCACTGCTTACTGGCTTATCGA

AATTAATACGACTCACTATAGGGAGACCCAAGCTG

>hiY Chloride-sensitive YFP (EYFP/ V163S A206K). Cloned into BstEII sites (NT13).

ggtgaccctaccgtcAGCAAGGGCGAGGAGCTGTTCACCGGGGTGGTGCCCATCCTGGTCGAGCTGGACGGCGACGTAAA

CGGCCACAAGTTCAGCGTGTCCGGCGAGGGCGAGGGCGATGCCACCTACGGCAAGCTGACCCTGAAGTTCATCTGCACCA

CCGGCAAGCTGCCCGTGCCCTGGCCCACCCTCGTGACCACCTTCGGCTACGGCCTGCAGTGCTTCGCCCGCTACCCCGAC

CACATGAAGCAGCACGACTTCTTCAAGTCCGCCATGCCCGAAGGCTACGTCCAGGAGCGCACCATCTTCTTCAAGGACGA

CGGCAACTACAAGACCCGCGCCGAGGTGAAGTTCGAGGGCGACACCCTGGTGAACCGCATCGAGCTGAAGGGCATCGACT

TCAAGGAGGACGGCAACATCCTGGGGCACAAGCTGGAGTACAACTACAACAGCCACAACGTTTATATCATGGCCGACAAG

CAGAAGAACGGCATCAAGTCGAATTTCAAGATCCGCCACAACATCGAAGACGGCAGCGTGCAGCTCGCCGACCACTACCA

GCAGAACACCCCCATCGGCGACGGCCCCGTGCTGCTGCCCGACAACCACTACCTGAGCTACCAGTCCAAACTGAGCAAAG

ACCCCAACGAGAAGCGCGATCACATGGTCCTGCTGGAGTTCGTGACCGCCGCCGGGATCACTCTCGGCATGGACGAGCTC

TACAAGggtgacc

>CFP mCerulean. Cloned into BsteII sites (in NT15).

ggtgaccctaccgtcAGCAAGGGCGAGGAGCTGTTCACCGGGGTGGTGCCCATCCTGGTCGAGCTGGACGGCGACGTAAA

CGGCCACAAGTTCTCGGTCTCTGGCGAGGGCGAGGGCGATGCCACCTACGGCAAGCTGACCCTGAAGTTCATCTGCACCA

CCGGCAAGCTGCCCGTGCCCTGGCCCACCCTCGTGACCACCCTGACCTGGGGCGTGCAGTGCTTCGCTCGCTACCCCGAC

CACATGAAGCAGCACGACTTCTTCAAGTCCGCCATGCCCGAAGGCTACGTCCAGGAGCGCACCATCTTCTTCAAGGACGA

CGGCAACTACAAGACCCGCGCCGAGGTGAAGTTCGAGGGCGACACCCTGGTGAACCGCATCGAGCTGAAGGGCATCGACT

TCAAGGAGGACGGCAACATCCTGGGGCACAAGCTGGAGTACAACGCCATCAGCGACAACGTTTATATCACCGCCGACAAG

CAGAAGAACGGCATCAAGGCCAACTTCAAGATCCGCCACAACATCGAGGACGGCAGCGTGCAGCTCGCCGACCACTACCA

GCAGAACACCCCCATCGGCGACGGCCCCGTGCTGCTGCCCGACAACCACTACCTGAGCACCCAGTCCAAACTGAGCAAAG

ACCCCAACGAGAAGCGCGATCACATGGTCCTGCTGGAGTTCGTGACCGCCGCCGGGATCACTCTCGGCATGGACGAGCTC

TACAAGggtgacc

>3xHA tag. Alternate at N-terminus, NheI-BstEII. (In NT15 with CFP in pcDNA3.1hygro, not discussed here).

GCTAGCCCGGGCACGGCCGCCACCaTGggcTACCCCTACGACGTGCCCGACTACGCCGGCTACCCCTACGACGTGCCCGA

CTACGCCGGCTCCTACCCCTACGACGTGCCCGACTACggtgacc

>NT51 HpaI-BsmBI inserted in NT17. Mutates 11 T’s and 4 S’s in the N-terminus GTTAACTTCGTAGATCCTGCTGCCAGCAATTCCGCAGAAGATCAGCTGTCAGATGCCGCCGGGGTCGGAGTCGATGGACC

CAACGTGCAGTTCCAGAACGGCGGGGACGCTGTACTGAGCGAGGGCCGTAGCCTCCATTCCGGTGGCGGCGGCGGCGAGG

GCCACCACCAGCACTACTATTATGATGCTCACGCTAACCAGTACTATCTGCGCGCTTTCGGCCACAACGCTATGGACGCG

GTACCCAGGATCGACCACTACCGGCACGCTGCCGCGCAGCTCGGCGAGAAGCTGCTCCGGCCTAATCTGGCCGAGCTGCA

TGACGAGCTGGAAAAGGAACCTTTTGAGGATGGCTTTGCAAATGGGGAAGAATCTGCTCCACAGCGTGATGCTGTGGTCG

AGTACCGTGCCGAAAGTAAAGGAGTCGTGAAGTTTGGCTGGATCAAGGGTGTGCTAGTACGTTGTATGTTAAACATTTGG

GGTGTGATGCTTTTCATTCGTCTC

>NT931 Xba-BspEI inserted in NT17. Extracellular 2xHA epitope (ECL2).

TCTAGAAGTCTAGGGCCTGAATTTGGTGGTGCCATTGGTCTAATCTTCGCCTTTGCCAACGCTGTTGCTGTTGCTATGTA

TGTGGTTGGATTTGCAGAAACCGTGGTGGAGTTGCTGAAAGAATACCCTTACGATGTCCCTGACTACGCCGGCTACCCAT

ACGACGTGCCAGACTACGCTCATTCCATTCTTATGATAGATGAAATCAATGACATCCGGA

>NT933 XhoI-EcoRI inserted in NT17. Extracellular 2xHA epitope (ECL3).

CTCGAGAGCTACCCTTACGATGTCCCTGACTACGCCGGCTACCCATACGACGTGCCAGACTACGCTAAGAAGCCAAAAGG

GTTTTTTGGCTATAAATCTGAAATCTTTAATGAGAACTTTGGCCCCGATTTTCGAGAAGAAGAAACTTTCTTTTCTGTAT

TTGCCATCTTTTTTCCTGCTGCAACTGGAATTC

>NT935 BsiWI-SacII inserted in NT17. Extracellular 2xHA epitope (ECL4).

CGTACGCGATGCCACTGGTAATGTTAATGACACTATCGTAACAGAGCTAACAAACTGTACTAGCGCAGCCTGCAAATTAA

ACTTTGATTACCCTTACGATGTCCCTGACTACGCCGGCTACCCATACGACGTGCCAGACTACGCTTTTTCATCTTGTGAA

TCCAGTCCTTGTAGCTACGGCCTAATGAACAACTTTCAGGTAATGAGTATGGTGTCAGGATTTACACCACTAATTTCCGC

GG

>NT103 PmlI-BsmBI inserted in NT17. C295S in TM1.

CACGTGATGCTGTGGTCACGTACACGGCCGAAAGTAAAGGAGTCGTGAAGTTTGGCTGGATCAAGGGTGTACTGGTACGT

TCTATGTTAAACATTTGGGGTGTGATGCTTTTCATTCGTCTCTCATGcgtctc

>NT104 PmlI-BsmBI inserted in NT17. C295A in TM1.

CACGTGATGCTGTGGTCACGTACACGGCCGAAAGTAAAGGAGTCGTGAAGTTTGGCTGGATCAAGGGTGTACTGGTACGT

GCCATGTTAAACATTTGGGGTGTGATGCTTTTCATTCGTCTCTCATGcgtctc

>NT360 Quickchange of NT17. C543M in TM7.

Primers gccgtgtctgtaggttctatggtcgtacgcgatgccact and agtggcatcgcgtacgaccatagaacctacagacacggc

>NT864 BsiWI-SacII inserted in NT17. ECL4 cysless. C563,568,577,582 to S

CGTACGCGATGCCACTGGTAATGTTAATGACACTATCGTAACAGAGCTAACAAACTCTACTAGCGCAGCCTCTAAATTAA

ACTTTGATTTTTCATCTTCTGAATCCAGTCCTTCTAGCTACGGCCTAATGAACAACTTTCAGGTAATGAGTATGGTCTCA

GGATTTACACCACTAATTTCCGCGG

>NT865 BsiWI-SacII inserted in NT17. ECL4 deletion removes C563,568,577,582.

CGTACGCGATGCCACTGGTAATGTTAATGACACTATCGTAACAGAGCTAACAAACAGCTACGGCCTAATGAACAACTTTC

AGGTAATGAGTATGGTCTCAGGATTTACACCACTAATTTCCGCGG

>NT399 SacII-PflMI inserted in NT17. C630A in TM8.

CCGCGGGCATATTCTCTGCCACTCTGTCTTCCGCATTAGCATCCCTAGTGAGTGCTCCCAAGATATTCCAGGCTCTAGCC

AAAGACAACATCTACCCCGCTTTCCAGATGTTTGCTAAAGGTTATGGGAAAAATAATGAGCCATTACGTGG

>NT400 SacII-PflMI inserted in NT17. C630S in TM8.

CCGCGGGCATATTCTCTGCCACTCTGTCTTCCGCATTAGCATCCCTAGTGAGTGCTCCCAAGATATTTCAGGCCCTATCC

AAAGACAACATCTACCCCGCTTTCCAGATGTTTGCTAAAGGTTATGGGAAAAATAATGAGCCATTACGTGG

>NT501 SwaI-BamHI inserted in NT17. C723S,C724V in TM11.

ATTTAAATACTACAATATGTGGATCTCACTTCTTGGAGCAATTCTTTCCGTGATAGTAATGTTCGTCATTAACTGGTGGG

CTGCTTTGCTAACCTACGTGATAGTCCTTGGTCTCTATATTTATGTTACCTACAAAAAACCCGATGTGAATTGGGGATCC

>NT572 BamHI-FseI inserted in NT501. C791S in Cterm, C723S,C724V in TM11.

GGATCCTCTACACAAGCCCTGACTTACCTCAATGCCCTGCAACATAGTATTAGGCTTTCTGGCGTGGAAGACCATGTGAA

AAACTTTAGGCCACAGTCCCTTGTTATGACAGGAGCGCCAAACTCACGGCCGGCC

>NT573 FseI-AfeI inserted in NT501. C820S in Cterm, C723S,C724V in TM11.

GGCCGGCCTTACTTCATCTTGTTCACGATTTCACAAAAAATGTTGGTTTGATGATCTCTGGCCACGTACACATGGGACCA

CGCAGACAAGCCATGAAAGAAATGTCCATCGACCAAGCCAAATATCAGCGCT

>NT674 PshAI-ClaI inserted in NT501. C1052S in Cterm, C723S,C724V in TM11.

GACGGAGGTCTCACATTACTCATACCTTACCTTCTGACGACCAAGAAAAAATGGAAAGACTCTAAGATCAGAGTATTCAT

TGGTGGAAAGATAAACAGAATAGACCATGACAGAAGAGCGATGGCTACTTTACTGAGCAAGTTCAGAATCGAT
